# Iron-Mediated Lysosomal Membrane Permeabilization in Ethanol-Induced Hepatic Oxidative Damage and Apoptosis: Protective Effects of Quercetin

**DOI:** 10.1155/2016/4147610

**Published:** 2015-12-28

**Authors:** Yanyan Li, Man Chen, Yanyan Xu, Xiao Yu, Ting Xiong, Min Du, Jian Sun, Liegang Liu, Yuhan Tang, Ping Yao

**Affiliations:** ^1^Department of Nutrition and Food Hygiene, School of Public Health, Tongji Medical College, Huazhong University of Science & Technology, 13 Hangkong Road, Wuhan 430030, China; ^2^Shenzhen Center for Chronic Disease Control, 2021 Buxin Road, Shenzhen, Guangdong 518020, China; ^3^Ministry of Education Lab of Environment and Health, School of Public Health, Tongji Medical College, Huazhong University of Science & Technology, 13 Hangkong Road, Wuhan 430030, China; ^4^Hubei Key Laboratory of Food Nutrition and Safety, School of Public Health, Tongji Medical College, Huazhong University of Science & Technology, 13 Hangkong Road, Wuhan 430030, China

## Abstract

Iron, in its free ferrous states, can catalyze Fenton reaction to produce OH∙, which is recognized as a crucial role in the pathogenesis of alcoholic liver diseases (ALD). As a result of continuous decomposition of iron-containing compounds, lysosomes contain a pool of redox-active iron. To investigate the important role of intralysosomal iron in alcoholic liver injury and the potential protection of quercetin, male C57BL/6J mice fed by Lieber De Carli diets containing ethanol (30% of total calories) were cotreated by quercetin or deferoxamine (DFO) for 15 weeks and ethanol-incubated mice primary hepatocytes were pretreated with FeCl_3_, DFO, and bafilomycin A1 at their optimal concentrations and exposure times. Chronic ethanol consumption caused an evident increase in lysosomal redox-active iron accompanying sustained oxidative damage. Iron-mediated ROS could trigger lysosomal membrane permeabilization (LMP) and subsequent mitochondria apoptosis. The hepatotoxicity was attenuated by reducing lysosomal iron while being exacerbated by escalating lysosomal iron. Quercetin substantially alleviated the alcoholic liver oxidative damage and apoptosis by decreasing lysosome iron and ameliorating iron-mediated LMP, which provided a new prospective of the use of quercetin against ALD.

## 1. Introduction

Alcoholic liver disease (ALD) is a primary consequence of heavy and prolonged drinking and represents a major cause of morbidity and mortality worldwide [1]. It is now well accepted that the pathogenesis of ALD is multifactorial, and iron-mediated oxidative stress has attracted emerging attention [2]. Alcohol stimulates iron absorption and hepatic storage by inhibiting hepcidin secretion, which initiates “free” iron release from various iron-containing proteins [3]. Cellular “free” iron, present as labile iron pool (LIP), is low-molecular weight, redox-active, and weakly chelated iron [4]. In case of excess, such redox-active isoform of iron may synergistically and dramatically amplify ethanol-induced oxidative stress and then exacerbate alcoholic liver injury by serving as a catalyst for hydroxyl radical (^∙^OH, the most toxic fraction among various reactive oxygen species (ROS)) formation via Fenton/Haber-Weiss reaction [5].

Lysosomes are responsible for autophagic turnover of all organelles and most long-lived proteins. As a consequence of degradation of many iron-containing macromolecules (e.g., ferritin and mitochondrial components), lysosomal compartments can accumulate large amounts of low mass redox-active iron and, therefore, enhance susceptibility to oxidative stress [6, 7]. Moreover, the ongoing digestion of such metalloproteins is accompanied by the release of redox-active iron which, upon export from the lysosome, may be a major intracellular source of LIP. In our previous work, we considered that mucolipin 1 mediated the release of endosomes and lysosome iron contributed to the increased LIP induced by ethanol [8]. Additionally, lysosomes are intimately associated with apoptotic cell death. Iron-mediated lysosomal ROS generation causes lysosomal membrane permeabilization (LMP) [9] and the ensuing release of cathepsins into the cytosol and thereby induces apoptosis through activation of mitochondrial membrane permeabilization (MMP) [10]. Growing studies have demonstrated that intralysosomal iron is a key determinant of ROS-induced injury [11, 12], and LMP has been presumed as an early event of lysosome-mitochondrial apoptosis pathway based on the blocking effect of deferoxamine (DFO) which is a typical lysosomal targeted iron chelator [13]. However, it still remained unclear, to our knowledge, what the role of intralysosomal redox-active iron is upon ethanol-induced hepatic oxidative damage and apoptosis.

In spite of common use for iron chelation therapy, DFO exerts the main limitation because of its potential toxicity and parenteral administration by slow subcutaneous injection with poor patient compliance [14]. Thus, there is an accumulating interest in the natural iron chelators originated from dietary phytochemicals. Quercetin, a naturally occurring bioflavonoid widely distributed in vegetables, fruits, and herb medicine, scavenges free radicals and exhibits iron-binding activity dependent on its structure characterized as phenolic hydroxyl groups [15]. We found that quercetin evidently attenuates ethanol-derived damage as a result of its prominent antioxidative properties [16]. Quercetin has been also demonstrated to significantly decrease hepatic iron and effectively quench intracellular free iron-induced ^∙^OH production [17]. Furthermore, quercetin supplementation modulated lysosomal enzyme activities in various diseases [18, 19] suggesting that the hepatoprotective effect of quercetin against alcohol-derived injury might be involved in the regulation and chelation of quercetin on lysosomal labile iron. Therefore, we hypothesized that lysosome redox-active iron may play an important role in ethanol-induced liver damage and quercetin potentially relieves ethanol-elicited hepatic oxidative damage and apoptosis by decreasing intralysosomal redox-active iron-mediated ROS overproduction and the subsequent LMP. Hence, we designed this study to probe the role of intralysosomal low mass iron toxicity in the process of ALD and the protection of quercetin.

## 2. Material and Methods

### 2.1. Chemicals and Reagents

Quercetin (purity ≥ 95%) and desferrioxamine (DFO) were from Sigma (USA). Collagenase (type IV), silver-lactate, bafilomycin A1, acridine orange (AO), and tetramethylrhodamine methyl ester (TMRM) were purchased from Sigma-Aldrich (USA). Dulbecco's Modified Eagle's Medium (DMEM) was obtained from Gibco (USA). In situ cell death detection kit was provided by Roche (Switzerland). Ethanol and FeCl_3_ were purchased from Zhenxing Chemical Factory (Shanghai, China). Dihydroethidium (DHE), 2′,7′-Dichlorodihydrofluorescein diacetate (H_2_DCFDA), and LysoTracker Red were purchased from Beyotime Corporation (Jiangsu, China). Cathepsin B activity assay kit and apoptosis assay kit were from BioVision (USA) and KenGEN BioTECH (Nanjing, China), respectively. GSH/GSSH assay kit was purchased from the Nanjing Jiancheng Corp., China. Cleaved caspase-3 antibody and cytochrome C antibody were purchased from CST (USA). Cathepsin B antibody and rabbit ferritin antibody were from Santa Cruz (USA) and Abcam (UK), respectively. Mouse monoclonal antibody GAPDH was obtained from Wuhan Boster Corp. (China). Other reagents used were of the purest grade available.

### 2.2. Animal Treatment

Animals were cared for according to the Guiding Principles in the Care and Use of Laboratory Animals published by the US National Institutes of Health. The male C57BL/6J mice (18~20 g), obtained from Sino-British Sippr/BK (Shanghai, China), were pair-fed with either regular or ethanol-containing Lieber De Carli liquids diets (Beijing HFK Bioscience Co., Ltd., Beijing, China) for 15 weeks. Following 1 week of acclimation, the mice were divided randomly into six groups of fifteen each: (1) normal control group (C), (2) ethanol group (E, 30% of total calories as ethanol), (3) ethanol plus quercetin group (EQ, quercetin: 100 mg/kg.bw), (4) quercetin control group (Q), (5) ethanol plus DFO group (ED, DFO: 100 mg/kg.bw), (6) DFO control group (D). Quercetin and DFO were received by gavage and intraperitoneal injections, respectively, according to the body weight. After 15 weeks, hepatocytes were isolated from three mice of each group to assay lysosomal status and intralysosomal iron content. Other mice were sacrificed after an overnight fasting and hepatic samples were collected rapidly for further biochemical assays.

### 2.3. Primary Hepatocytes Isolation and Treatment

According to a two-step collagenase described by Figliomeni and Abdel-Rahman [20], primary hepatocytes were isolated from adult male C57BL/6J mice (22~25 g). The freshly harvested hepatocytes (≥90% viability) were suspended in DMEM medium with 10% fetal bovine serum, 100 U/mL penicillin, 100 U/mL streptomycin, 32 IE/L insulin, 0.1 *μ*mol/L hydrocortisone, and 15 mol/L HEPES and then inoculated in rat tail collagen-coated 6-well plates for culture in a humidified incubator at 37°C, 5% CO_2_. On the following day, the attached hepatocytes were incubated with medium containing various pharmacological reagents. Before subjecting to ethanol (100 mmol/L), some cultures were exposed to bafilomycin A1 (100 nmol/L), FeCl_3_ (60 *μ*mol/L), DFO (1 mmol/L), and quercetin (100 *μ*mol/L). After 24 h, the supernatant and cells were collected for various bioassays according to the corresponding experimental protocol. The optimal concentration and exposure times of the all regents were established in preliminary experiments.

### 2.4. Measurement of Hepatic Redox Status

Malondialdehyde (MDA) was measured by using thiobarbituric acid colorimetry slightly modified by Ohkawa et al. [21]. Reduced glutathione (GSH) and oxidized glutathione (GSSH) were measured with the assay kit. The MDA level was standardized by protein concentration evaluated with the Bio-Rad protein assay kit (USA).

DHE was used for in situ determination of ROS in mice liver tissues. The frozen hepatic sections (5 *μ*m) were immediately incubated with DHE (5 *μ*M) at 37°C for 15 min. Then the red fluorescence intensity excited by green laser was visualized with a fluorescence microscope (Melville, NY). Fluorescence intensities in randomly selected areas of the images were quantified using IPP 6.0 image analysis software. ROS production in mice primary hepatocytes was assayed by adding H_2_DCFDA (10 *μ*M) to the culture medium for 30 min, followed by the quantifications with spectrometry (Ex: 485 nm, Em: 525 nm).

### 2.5. Assay for Detecting Culture Medium Biomarkers for Liver Injury

Levels of medium aspartate (AST), alanine aminotransferases (ALT), and lactate dehydrogenase (LDH) were measured with enzymatic kinetic method by Mindray BS-200 automatic biochemistry analyzer (Shenzhen, China).

### 2.6. Apoptosis Assay

Apoptotic cells were identified in formalin-fixed, paraffin-embedded liver tissue sections, with the classic in situ Dead End TM fluorometric TdT-mediated dUTP nick end labeling (TUNEL) assay according to the manufacturer's protocol. The expression of cleaved caspase-3 was measured by Western blot and caspase-3 activity was detected with the assay kit (Beyotime, China) according to manufacturer's instructions. In cell experiment, the apoptosis cells were measured by flow cytometryusing annexin V/PI assay kit.

### 2.7. Assay of Hepatic Total Iron, LIP, and Lysosomal Low Mass Reactive Iron

Total amount of hepatic iron was determined by flame atomic absorption spectrometry (spectrAA-240FS, USA) using an iron hollow cathode lamp. The analysis of LIP was performed according to Khan et al. [22]. In brief, liver homogenates (2.5%) were prepared using 1 mM ethylenediaminetetraacetic acid (EDTA) to dissociate LIP and centrifuged at 20,000 ×g for 15 min at 4°C. The resultant supernatants were ultra-filtrated on Micron-30 at 14,000 ×g for 20 min at 4°C for atomic absorption spectrophotometric assay as LIP.

For detection of lysosomal low mass reactive iron content, we used the modified sulfide-silver method (SSM) [23, 24]. In brief, hepatocytes were grown on coverslips and were then washed with PBS and fixed with 2% glutaraldehyde in 0.1 M Na-cacodylate (Amresco, USA) buffer with 0.1 M sucrose (pH = 7.2) for 2 h at 22°C. Next cells were sulfidated at pH ≈ 9 with 1% (w/v) ammonium sulphide (Zhenxing, China) in 70% (v/v) ethanol for 15 min. The development was then performed in the dark at 26°C for 20~50 min using a physical, colloid-protected developer containing silver-lactate (Sigma, USA). Following hydroquinone in a graded series of ethanol solutions and mounting in Canada balsam (Ourchem, China), the cells were examined with microscope.

### 2.8. Vital Imaging of Lysosomal ROS

Hepatocytes were coincubated with H_2_DCFDA (10 *μ*M) and LysoTracker Red (100 nM) for 30 min, and images were sequentially collected in the green and red channels with the fluorescence microscope and merged with IPP 6.0 software.

### 2.9. Analysis of Lysosomal Membrane Permeabilization (LMP)

LMP was evaluated by acridine orange (AO), a lysosomotropic base containing metachromatic fluorophore, which becomes charged and retained within acidic compartments [25]. Hepatocytes were stained with AO (5 *μ*g/mL) in complete medium at 37°C for 15 min. AO-induced red (lysosomal) and green (nuclear and cytosolic) fluorescence were observed using fluorescence microscope (Melville, NY). LMP was also measured by flow cytometry. The green (FL1) and red (FL3) fluorescence of 10,000 cells was recorded on a logarithmic scale using a Becton Dickinson FACScan instrument while excited at 488 nm (argon laser). Using this technique, the alterations of lysosomal stability were assayed, as evident by decrease in FL3 red fluorescence. With the alteration of LMP, we measured lysosomal cathepsin B relocation and activity by Western blot and assay kit, respectively.

### 2.10. Analysis of Mitochondrion Membrane Permeabilization (MMP)

Cells were incubated with 100 nM TMRM for 30 min at room temperature and washed with PBS, and red mitochondrial fluorescence of 10,000 cells per sample was determined by flow cytometry by using the FL3 channel. Cells with increased MMP showed decreased TMRM red fluorescence.

### 2.11. Separation of Subcellular Fractions

The fresh liver tissue samples were washed in PBS to remove blood and then were homogenized in 0.25 M ice cold sucrose solution [26]. The homogenate was subjected to differential centrifugation for different fractions: (1) structural proteins, nucleus, and cell debris, 600 g, 10 min; (2) mitochondria, 5000 g, 10 min; (3) lysosomes, 15000 g, 10 min; (4) supernatant, cytosol.

### 2.12. Western Blot Analysis

Mice liver tissues were homogenized and lysed at 4°C in RIPA buffer and the lysates with equal amount of proteins were subjected to Western blot. The protein was separated by 10% SDS-polyacrylamide gel and then transferred onto a PVDF membrane. After incubation with the specific primary antibodies against the target protein and the species-specific second antibodies conjugated to horseradish peroxidase, immunoreactive bands were detected by means of an ECL plus Western Blotting Detection System (Amersham Biosciences, Little Chalford, UK). The band densities were measured by Image J software.

### 2.13. Statistical Analysis

Results are given as the mean ± standard deviation (SD) from replicate experiments and subjected to one-way ANOVA followed by Student-Newman-Keuls multiple range test (SPSS 16.0 software package). The statistical significance was defined as a difference between groups of *P* < 0.05.

## 3. Results

### 3.1. The Effect of Quercetin Supplementation on Body Weight and Liver Index of Mice Fed with Ethanol

As shown in Table 1, neither quercetin nor DFO had influence on final body weight and liver ratio against body weight (liver index) in comparison with normal control at the end of experimental periods. However, the weight gain of all ethanol-fed mice was lower than those of non-ethanol-challenged groups, and ethanol intake led to the marked increase in the liver index, whereas quercetin suppressed these adverse effects. Interestingly, DFO had no influence on mice body weight gain compared with ethanol group, while DFO normalized the increased liver index delivered by ethanol.

### 3.2. Quercetin and DFO Attenuated the Mice Liver Oxidative Damage Induced by Chronic Ethanol Consumption

**Figure 1:**
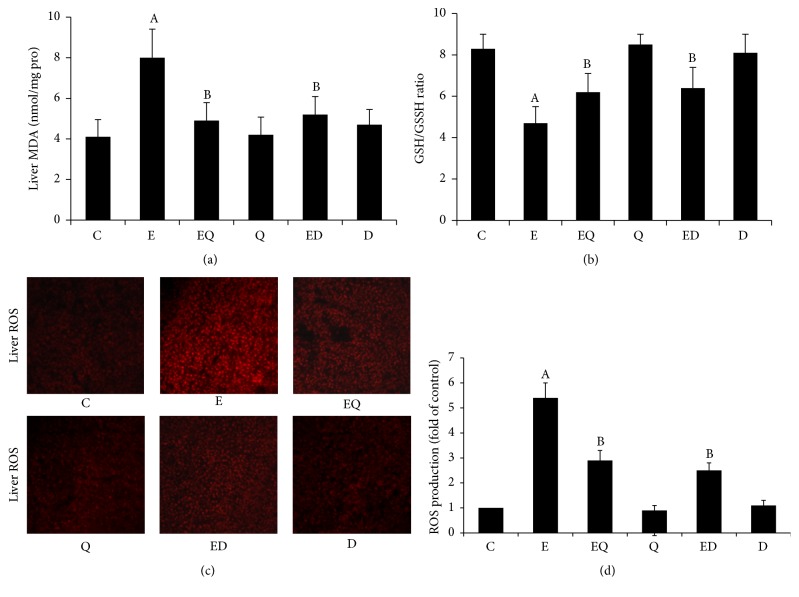
The effect of quercetin and DFO on the mice liver oxidative damage delivered by chronic alcohol consumption. The liver MDA (a) and the ratio of GSH/GSSH (b) were detected by spectrophotometry. Each value represents the mean ± SD (*n* = 12). Liver ROS (c) was detected and visualized with fluorescence microscope (×100). Fluorescence intensities in randomly selected areas of the images were quantified with IPP 6.0 image analysis software (d). C: control; E: ethanol; EQ: ethanol plus quercetin; ED: ethanol plus DFO; Q: quercetin; D: DFO. A: *P* < 0.05 versus the control; B: *P* < 0.05 versus the ethanol group.

As depicted in Figure 1, long-time ethanol treatment substantially provoked mice liver oxidative damage manifested by a remarkable MDA elevation, GSH depletion, and overproduction of ROS. As expected, both quercetin and DFO evidently normalized GSH level, decreased MDA content, and suppressed ROS generation derived from ethanol. However, quercetin and DFO per se had no influence on hepatic redox status compared with normal mice.

### 3.3. The Protective Effect of Quercetin and DFO against Ethanol-Induced Hepatic Apoptosis

**Figure 2:**
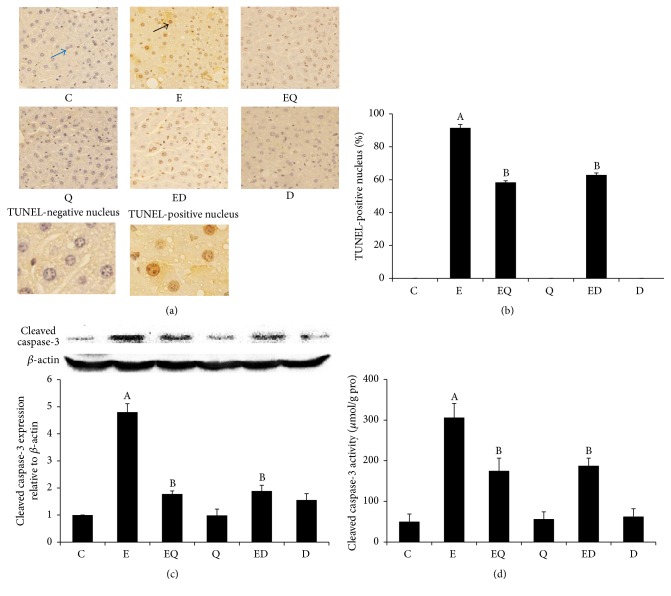
Quercetin extenuated hepatocytes apoptosis elicited by ethanol. TUNEL staining was used to assay hepatocytes apoptosis (a). The TUNEL-positive nucleus showed brown (black arrow) and the negative nucleus showed blue (blue arrow). (b) Quantitation of data obtained in (a) by using the IPP image analysis software. The activation of caspases-3 was analyzed by Western blotting (c, *n* = 3) and quantified by Image J software (c). Blotting with anti-*β*-actin was used as a protein loading control. The activity of caspase-3 was determined by assay kit (Beyotime) and standardized by protein concentration measured by the Bio-Rad protein assay kit (d). Results were presented as mean ± SD or mean ± SE. C: control; E: ethanol; EQ: ethanol plus quercetin; ED: ethanol plus DFO; Q: quercetin; D: DFO. A: *P* < 0.05 versus the control; B: *P* < 0.05 versus the ethanol group.

In situ apoptosis in mice liver section was evaluated by TUNEL staining. In the control group, quercetin group, and DFO group, TUNEL-positive cells were not observed. However, the number and signal density of TUNEL-positive hepatocytes significantly increased in the ethanol-exposed mice (Figures 2(a) and 2(b)). Enhanced protein of the apoptotic marker cleaved caspase-3 confirmed this finding, with a 4.8-fold increase in ethanol group compared to normal control (Figure 2(c)). In addition, long-time ethanol consumption increased caspase-3 activity (Figure 2(d)). Quercetin and DFO treatment apparently reduced the reactivity and the number of apoptotic hepatocytes compared with the ethanol group. And the expression of cleaved caspase-3 and caspase-3 activity were significantly decreased in quercetin and DFO treatment group.

### 3.4. Quercetin, Similar to DFO, Suppressed the Ethanol-Induced Iron Abnormity of Hepatic Tissue and Especially Lysosomal Compartment

**Figure 3:**
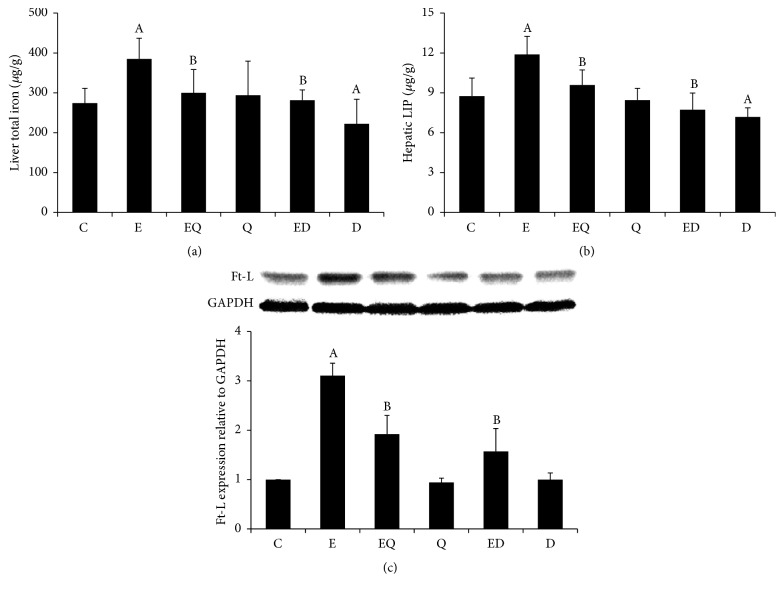
Quercetin decreased liver total iron and labial iron pool (LIP) as well as DFO. Mice liver total iron (a) and LIP (b) were determined by flame atomic absorption spectrophotometry. Each value represents the mean ± SD (*n* = 12). Ft-L expression was measured by Western blotting and the band densities were measured by Image J software (c, *n* = 3). C: control; E: ethanol; EQ: ethanol plus quercetin; ED: ethanol plus DFO; Q: quercetin; D: DFO. A: *P* < 0.05 versus the control; B: *P* < 0.05 versus the ethanol group.

**Figure 4:**
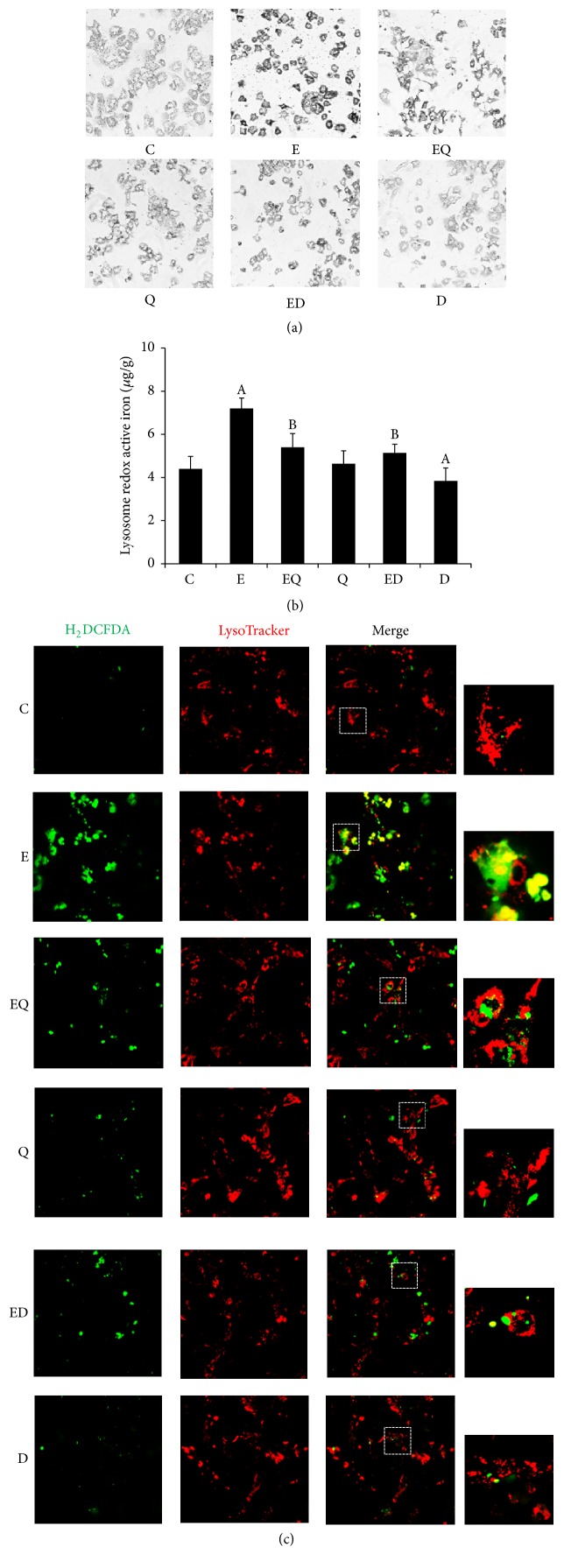
Quercetin decreased intralysosomal redox-active iron and it induced ROS in lysosome as well as DFO. The mice primary hepatocytes were isolated according to a two-step collagenase method and treatment for 6 hours. The intralysosomal iron was measured by SSM ((a) the exposure time was 50 min in the dark, *n* = 3) and quantitated by flame atomic absorption spectrophotometry (b). The cells were incubated with a combination of LysoTracker (to detect lysosomes) and H_2_DCFDA (to detect ROS production) for 30 min and immediately observed by confocal microscopy with sequential recording in green (oxidized product of H_2_DCF) and red (LysoTracker). All photomicrographs were taken at ×200 (c, *n* = 3). C: control; E: ethanol; EQ: ethanol plus quercetin; ED: ethanol plus DFO; Q: quercetin; D: DFO. A: *P* < 0.05 versus the control; B: *P* < 0.05 versus the ethanol group.

The hepatic contents of total iron and LIP were measured by flame atomic absorption spectrophotometry. As illustrated in Figures 3(a) and 3(b), chronic ethanol feeding gave rise to intracellular accumulation of iron in liver. 1.4-fold and 1.5-fold increase of liver total iron content and LIP compared to normal control were observed. And the expression of ferritin light chain (Ft-L) was upregulated (Figure 3(c)). Further, the intralysosomal redox-active iron was determined using SSM. Compared with normal control, the intralysosomal iron (Figures 4(a) and 4(b)) was significantly increased in ethanol-exposed mice with a distinct lysosomal silver precipitate accompanying the high iron content and hepatic LIP. The abnormal increases of both hepatic and lysosomal iron were partially reversed by quercetin or DFO supplementation. Interestingly, quercetin per se had no influence on hepatic total iron content, LIP, and lysosome iron level, while DFO treatment resulted in manifest decrease of liver iron comparing to normal control. Concomitant with the increase of intralysosomal redox-active iron, ROS largely colocalized with lysosomes. Quercetin decreased the lysosomal ROS generation as well as DFO (Figure 4(c)).

### 3.5. Quercetin and DFO Ameliorated LMP and MMP Delivered by Ethanol

**Figure 5:**
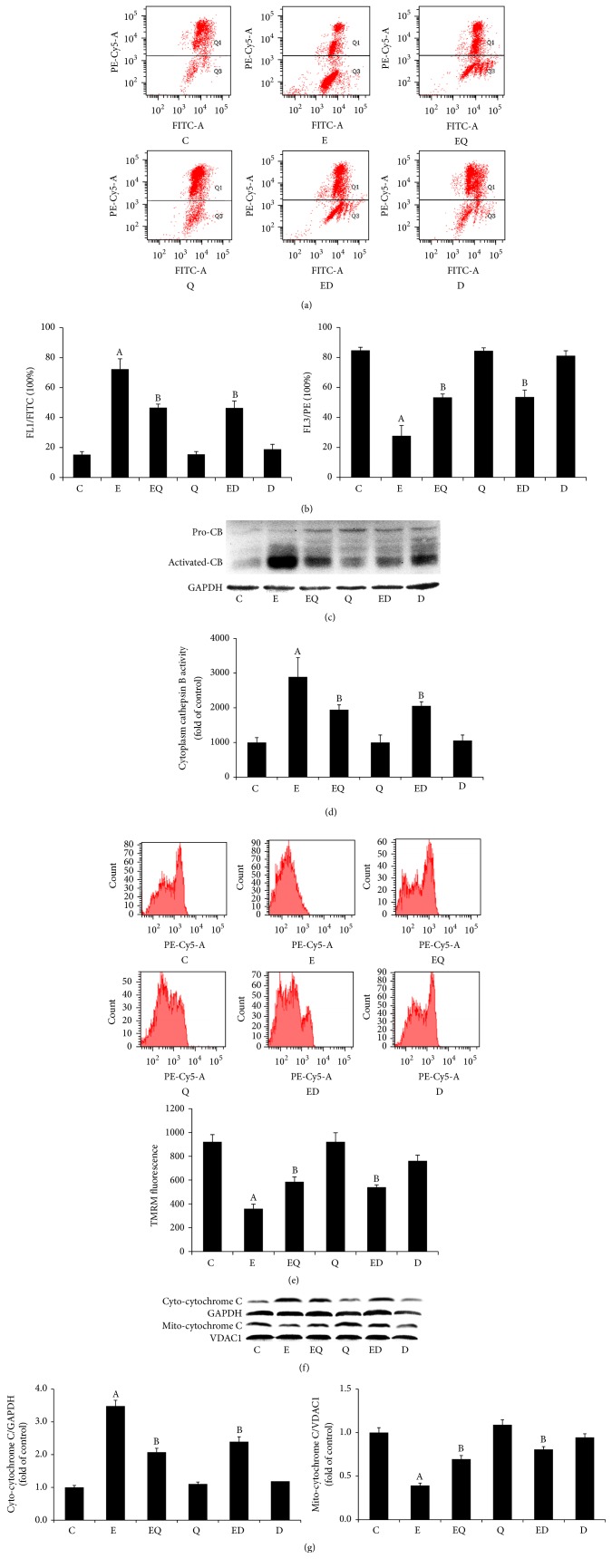
Quercetin attenuated ethanol-induced lysosomal membrane permeabilization (LMP) and mitochondria membrane permeabilization (MMP). The alterations of LMP were assayed by AO staining with flow cytometry, as evident by decrease in FL3 red fluorescence and increase in FL1 green fluorescence (a and b). Cathepsin B reconstitution (c) and activity (d) were determined by Western blot and assay kit. The red mitochondrial fluorescence stained with TMRM of 10,000 cells per sample was determined by flow cytometry by using the FL3 channel (e). Cytochrome C release (f) was evaluated by Western blotting and the band densities were measured by Image J software (g, *n* = 3). The blotting with GAPDH and VDAC1 was used as a protein loading control. C: control; E: ethanol; EQ: ethanol plus quercetin; ED: ethanol plus DFO; Q: quercetin; D: DFO.

Excess intralysosomal iron-catalyzed ROS may cause LMP, whereby lysosomal cathepsins like cathepsin B are released from the lysosomal lumen to the cytosol, and resultantly trigger MMP. As expected, in contrast with the normal control, chronic ethanol consumption caused serious LMP manifested as bright green fluorescence in FL1 and weakened red fluorescence in FL3 (Figures 5(a) and 5(b)). Upon LMP, lysosomal cathepsin B is released to the cytosol and activated. We also found that the cytosolic CB activity increased significantly (Figures 5(c) and 5(d)). Besides, TMRM staining revealed a significant increase of MMP in ethanol-fed mice hepatocytes compared with normal control (Figure 5(e)) and subsequent release of cytochrome C from mitochondria to cytoplasm (Figures 5(f) and 5(g)). Quercetin markedly ameliorated the ethanol-triggered LMP evidenced by increased red fluorescence. Subsequent disorders, including cathepsin B release, MMP, and cytochrome C release, were also partially normalized as a result of quercetin regime to ethanol-fed mice.

### 3.6. The Role of Lysosomal Iron in Ethanol-Induced Hepatocytes Damage and the Protective Effect of Quercetin

**Figure 6:**
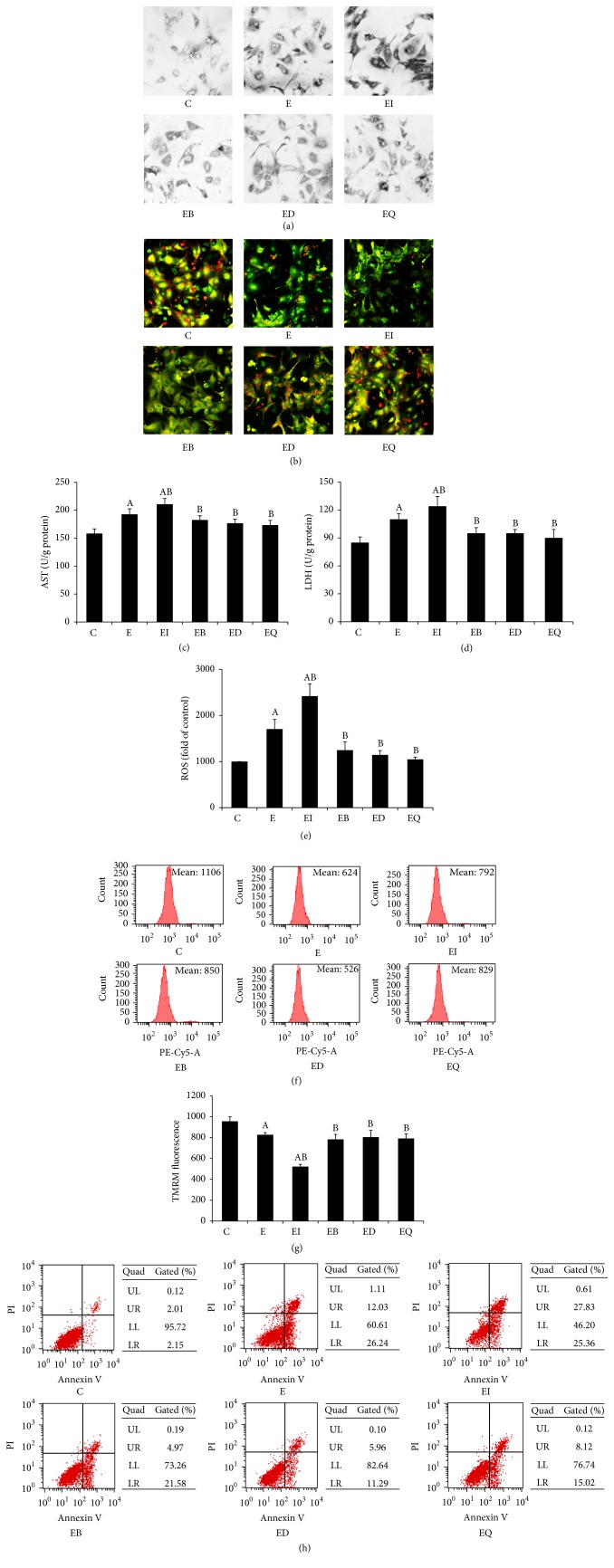
The role of lysosomal iron in ethanol-induced hepatocytes damage and the protective effect of quercetin. Mouse primary hepatocytes grown on coverslips were treated with ethanol (100 mmol/L, 24 hours), bafilomycin A1 (100 nmol/L, 4 hours), FeCl_3_ (60 *μ*mol/L, 4 hours), DFO (1 mmol/L, 4 hours), and quercetin (100 *μ*mol/L, 24 hours). The intralysosomal redox-active iron content (a) and lysosome status (b) were measured by SSM (the exposure time for the FeCl_3_ group was 20 min; other groups were 50 min) and AO-uptake technique, respectively. All photomicrographs were taken at ×200 (*n* = 3). The leakage of AST and LDH from hepatocytes (c, d, *n* = 12), together with cellular ROS production (e, *n* = 12), was detected by spectrophotometry. MMP (f and g) and apoptosis (h) were measured by flow cytometry (*n* = 3). Each value represents the mean ± SD. C: control; E: ethanol; EI: ethanol plus FeCl_3_; EB ethanol plus bafilomycin A1; ED: ethanol plus DFO. A: *P* < 0.05 versus the control; B: *P* < 0.05 versus the ethanol group.

To further confirm the crucial role of intralysosomal redox-active iron in ethanol-elicited hepatic oxidative damage and apoptosis, mouse primary hepatocytes grown on coverslips were incubated with bafilomycin A1, FeCl_3_, and DFO before exposing to ethanol. The intralysosomal reactive iron content and lysosome status were measured by SSM and AO-uptake technique, together with cellular injury assay. As shown in Figure 6, compared with normal intact hepatocytes, ethanol markedly increased lysosomal silver precipitation, indicting a substantial accumulation of lysosomal redox-active iron. Moreover, ethanol incubation decreased the lysosome stability evidenced by lowered red fluorescence of AO, caused ROS overproduction, increased MMP, caused apoptosis, and elevated the release of cellular AST and LDH. What is worse, the intralysosomal iron level of the hepatocytes treated with FeCl_3_ was higher than the ethanol group, and the excess iron increased further lysosomal destabilization and hepatic damage. This damage was blocked by bafilomycin A1 (bafilomycin A1 is a highly potent and selective vacuolar type H^+^-ATPase (V-ATPase) inhibitor that inhibits the acidification of lysosomes, thus blocking lysosome activity and degradation) or DFO (chelation of redox-active iron) by reducing the lysosome low mass reactive iron.

## 4. Discussion

Fenton reaction, a homolytic splitting of H_2_O_2_ yielding the extremely reactive hydroxyl radical (^∙^OH), may mainly take place inside the lysosomal compartment where iron occurs in its low mass redox-active form (be regarded as lysosome LIP) [27]. ^∙^OH mediated cell damage may be initiated by peroxidative injury to lysosomal membranes. Resulting relocation of low mass iron and cathepsins would lead to secondary harm to various cellular constituents. There were substantial evidences for the major determinant of lysosomal iron induced oxidative damage and apoptosis in various diseases including neurodegenerative diseases, atherosclerosis, and diabetes [28–30]. In our current study, chronic ethanol abuse caused intralysosomal iron accumulation concomitant with the increase of hepatic total iron and ferritin light chain. Consequently, liver oxidative damage and lysosome-mitochondrial apoptosis were triggered. Such detrimental outcomes were notably reversed by quercetin and DFO. Thus, the role of aberrant lysosome LIP level and lysosome damage in alcoholic liver injury was a focus of our investigations since iron chelation especially the intralysosomal redox-active iron chelation was assumed as a new plausible strategy to ameliorate ALD.

DFO, the common lysosomal target iron chelation, may accumulate within lysosomes and cause iron starvation with further negative consequences for iron metabolism [31, 32]. In this work, as a result of long-time DFO intraperitoneal injection, the mice liver iron was significantly lower than normal control, which may disturb liver iron metabolism. Excitingly, plant-derived quercetin was shown to be a potent iron chelator similarly active to DFO, which, at least partly, contributes to its antioxidative properties [15, 33]. Moreover, quercetin could readily permeate cell membranes via glucose transport proteins (GLUTs) [34] to quench intracellular ^∙^OH as a shuttle for labile iron [35, 36]. As we expected, quercetin manifested an extensive protection against ethanol stimulated abnormal intralysosomal LIP level and the ensuing liver oxidative damage and apoptosis. It has been proposed that ferritin-iron release requires lysosomal activity, which is a major source of lysosome redox-active iron [37]. Ferritin is a main cellular iron-binding protein and its light chains (Ft-L) were mainly expressed in liver which may contain large amounts of iron [38]. Our data showed that Ft-L expression was obviously upregulated by ethanol accompanying the high hepatic iron content, to some extent, by a self-protection mechanism. Moreover, some studies indicated that ferritin seems to be capable of binding intralysosomal iron temporarily to protect against iron-mediated oxidation when it is auto-phagocytosed [11]. In spite of this, we still believed that the recycling of ferritin-iron in lysosomal compartment was the major source of intralysosomal redox-active iron since the iron-binding capacity of ferritin was weak in acidic lysosomal lumens [39]. Quercetin partially normalized ferritin expression, which may contribute to its hepatoprotective effect by reducing intralysosomal redox-active iron.

LMP is considered to be a main mechanism of lysosome iron-induced damage [40]. Induction of LMP by ROS production has been extensively studied in model systems in which the intralysosomal iron content has been manipulated [41]. In our study, with the increasing lysosome iron, long-time ethanol consumption initiated ROS overproduction especially in lysosomes (colocalization with LysoTracker) and trigged serious LMP. It is admitted that LMP can cause iron release to cytoplasm directly and consequent prooxidant cell damage [42, 43]. What is worse, other lysosomal contents (e.g., H^+^, proteases) also leak which in turn accelerates the labile iron release from cytoplasmic ferritin to LIP [7, 39]. Here, our data showed that ethanol-elicited hepatic abnormal LIP was blocked by quercetin and we attributed repairing LMP to the positive effect. Evidence showed that the efflux of iron from the UVA-damaged lysosome was a major source of LIP in cells and epicatechin (one of the flavonoids) suppressed UVA-mediated release of labile iron by alleviating lysosomal rupture and ferritin degradation [44, 45]. Besides, LMP is emerging as an important regulator of cell apoptosis. LMP allows the release of cathepsin B, an apoptotic mediator, into the cytoplasm, where it can initiate the intrinsic apoptotic pathway [46]. Under normal physiological condition, cathepsin B is sequestered into the lysosomes of intact cells to participate in normal turnover of proteins [47]. Once into the cytoplasm, cathepsin B can cleave to activate proapoptotic proteins, including Bid [48], followed by engaging MMP and release of cytochrome C [49]. Thereafter, the release of cytochrome C causes the activation of effector caspases and triggers a caspase-dependent apoptotic pathway [50]. Our results suggested that chronic ethanol consumption triggered lysosome-mitochondrial apoptosis pathway. Quercetin quenched iron incited lysosomal ROS and blocked LMP. Subsequently, quercetin not only inhibited the leakage of cathepsin B into cytoplasm but arrested all measured parameters of mitochondrial apoptosis pathway including MMP, the escape of cytochrome C from mitochondria, and caspase-3 activity.

To further probe the pathophysiological role of lysosomal iron in ethanol triggered hepatic injury and the protection of quercetin, we utilized some strategies to regulate intralysosomal redox-active iron pool. In neutral medium, FeCl_3_ could form insoluble iron complexes and enter the lysosomal compartment by endocytosis [12]. We identified that enhancing free lysosome iron by adding FeCl_3_ aggravated ethanol stimulated LMP and subsequently sharpened ROS-induced damage and MMP-mediated apoptosis. Such harmful results, conversely, were counteracted by lysosomal iron chelator DFO. Furthermore, preincubation with bafilomycin A1 to block lysosome function [10] ameliorated primary hepatocytes damage, suggesting that ethanol-induced hepatic oxidative injury and mitochondria apoptosis involved low mass iron-delivered lysosome rupture. Most importantly, the detrimental effects on hepatocytes triggered by ethanol were also hampered by quercetin supplementation. Renovating LMP accompanied with substantially decreasing hepatic intralysosomal redox-active iron is more likely explanation of the powerfully protective activity of quercetin. To our knowledge, it is the first time to report that redox-active lysosomal iron seemed to be chief reason for ALD development. Overall, it may be a new potential strategy to attenuate alcoholic liver injury by reducing lysosomal redox-active iron and the ensuing LMP.

## 5. Conclusion 

All these findings strongly support the central role of a high amount of redox-active lysosomal iron in ethanol-elicited liver oxidative damage and apoptosis. Lowering intralysosomal redox-active iron pool and consequently the mitigation of LMP may be the potential mechanism of the powerful protection against alcoholic liver damage of quercetin. Our overall results highlighted a novel potential prospective for the prevention of ALD by naturally occurring iron-chelating quercetin.

## Figures and Tables

**Table 1:** Effects of quercetin on body weights and liver ratio to weight in the mice chronically fed ethanol for 15 weeks.

	Initial weight (g)	Final weight (g)	Weight gain (g)	Liver ratio to weight %
C	18.4 ± 1.1	28.9 ± 1.1	10.7 ± 2.2	4.6 ± 0.6
E	18.3 ± 1.2	23.1 ± 2.1^a^	4.8 ± 1.8^a^	5.8 ± 0.5^a^
EQ	18.5 ± 1.2	25.4 ± 1.8^b^	6.9 ± 1.9^b^	4.9 ± 0.4^b^
Q	18.4 ± 1.1	29.0 ± 1.5	10.6 ± 2.1	4.6 ± 0.3
ED	18.3 ± 1.1	24.1 ± 2.1	5.9 ± 1.9^b^	5.3 ± 0.4^b^
D	18.4 ± 1.1	26.8 ± 1.6	8.4 ± 1.7	4.7 ± 0.5

The male C57BL/6J mice were pair-fed with either regular or ethanol-containing Lieber De Carli liquids diets for 15 weeks. Quercetin and DFO were received by gavage and intraperitoneal injections, respectively, according to the body weight. Data were shown as mean ± SD, *n* = 12. ^a^ *P* < 0.05 versus control; ^b^ *P* < 0.05 versus ethanol. C: control; E: ethanol; EQ: ethanol plus quercetin; ED: ethanol plus DFO; Q: quercetin; D: DFO.

## Abbreviations

GSH: Reduced glutathione
MDA: Malondialdehyde
AST: Aspartate aminotransferases
ALT: Alanine aminotransferases
LDH: Lactate dehydrogenase
ROS: Reactive oxygen species
LIP: Labile iron pool
LMP: Lysosomal membrane permeabilization
MMP: Mitochondrion membrane permeabilization
TUNEL: Dead End TM fluorometric TdT-mediated dUTP nick end labeling.

## References

[1] Dey A., Cederbaum A. I. (2006). Alcohol and oxidative liver injury. *Hepatology*.

[2] Kohgo Y., Ohtake T., Ikuta K. (2005). Iron accumulation in alcoholic liver diseases. *Alcoholism: Clinical and Experimental Research*.

[3] Bridle K. R., Cheung T.-K., Murphy T. L. (2006). Hepcidin is down-regulated in alcoholic liver injury: implications for the pathogenesis of alcoholic liver disease. *Alcoholism: Clinical and Experimental Research*.

[4] Kruszewski M. (2003). Labile iron pool: the main determinant of cellular response to oxidative stress. *Mutation Research*.

[5] Bacon B. R., Britton R. S. (1990). The pathology of hepatic iron overload: a free radical-mediated process?. *Hepatology*.

[6] Kurz T., Terman A., Gustafsson B., Brunk U. T. (2008). Lysosomes in iron metabolism, ageing and apoptosis. *Histochemistry and Cell Biology*.

[7] Kurz T., Eaton J. W., Brunk U. T. (2011). The role of lysosomes in iron metabolism and recycling. *International Journal of Biochemistry and Cell Biology*.

[8] Tang Y., Li Y., Yu H. (2014). Quercetin attenuates chronic ethanol hepatotoxicity: implication of ‘free’ iron uptake and release. *Food and Chemical Toxicology*.

[9] Kurz T., Terman A., Brunk U. T. (2007). Autophagy, ageing and apoptosis: the role of oxidative stress and lysosomal iron. *Archives of Biochemistry and Biophysics*.

[10] Hamacher-Brady A., Stein H. A., Turschner S. (2011). Artesunate activates mitochondrial apoptosis in breast cancer cells via iron-catalyzed lysosomal reactive oxygen species production. *Journal of Biological Chemistry*.

[11] Kurz T., Terman A., Gustafsson B., Brunk U. T. (2008). Lysosomes and oxidative stress in aging and apoptosis. *Biochimica et Biophysica Acta—General Subjects*.

[12] Yu Z., Persson H. L., Eaton J. W., Brunk U. T. (2003). Intralysosomal iron: a major determinant of oxidant-induced cell death. *Free Radical Biology and Medicine*.

[13] Persson H. (2006). Radiation-induced lysosomal iron reactivity: implications for radioprotective therapy. *IUBMB Life*.

[14] Kattamis A., Ladis V., Berdousi H. (2006). Iron chelation treatment with combined therapy with deferiprone and deferioxamine: a 12-month trial. *Blood Cells, Molecules, and Diseases*.

[15] Leopoldini M., Russo N., Chiodo S., Toscano M. (2006). Iron chelation by the powerful antioxidant flavonoid quercetin. *Journal of Agricultural and Food Chemistry*.

[16] Yao P., Nussler A., Liu L. (2007). Quercetin protects human hepatocytes from ethanol-derived oxidative stress by inducing heme oxygenase-1 via the MAPK/Nrf2 pathways. *Journal of Hepatology*.

[17] Oliva J., Bardag-Gorce F., Tillman B., French S. W. (2011). Protective effect of quercetin, EGCG, catechin and betaine against oxidative stress induced by ethanol in vitro. *Experimental and Molecular Pathology*.

[18] Chougala M. B., Bhaskar J. J., Rajan M. G. R., Salimath P. V. (2012). Effect of curcumin and quercetin on lysosomal enzyme activities in streptozotocin-induced diabetic rats. *Clinical Nutrition*.

[19] Punithavathi V. R., Prince P. S. M. (2010). Pretreatment with a combination of quercetin and *α*-tocopherol ameliorates adenosine triphosphatases and lysosomal enzymes in myocardial infarcted rats. *Life Sciences*.

[20] Figliomeni M. L., Abdel-Rahman M. S. (1998). Ethanol does not increase the hepatotoxicity of cocaine in primary rat hepatocyte culture. *Toxicology*.

[21] Ohkawa H., Ohishi N., Yagi K. (1979). Assay for lipid peroxides in animal tissues by thiobarbituric acid reaction. *Analytical Biochemistry*.

[22] Khan M. F., Wu X., Alcock N. W., Boor P. J., Ansari G. A. S. (1999). Iron exacerbates aniline-associated splenic toxicity. *Journal of Toxicology and Environmental Health Part A: Current Issues*.

[23] Garner B., Roberg K., Brunk U. T. (1998). Endogenous ferritin protects cells with iron-laden lysosomes against oxidative stress. *Free Radical Research*.

[24] Zdolsek J. M., Roberg K., Brunk U. T. (1993). Visualization of iron in cultured macrophages: a cytochemical light and electron microscopic study using autometallography. *Free Radical Biology and Medicine*.

[25] Krolenko S. A., Adamyan S. Y., Belyaeva T. N., Mozhenok T. P. (2006). Acridine orange accumulation in acid organelles of normal and vacuolated frog skeletal muscle fibres. *Cell Biology International*.

[26] Prince P. S. M., Priscilla H., Devika P. T. (2009). Gallic acid prevents lysosomal damage in isoproterenol induced cardiotoxicity in Wistar rats. *European Journal of Pharmacology*.

[27] Berndt C., Kurz T., Selenius M., Fernandes A. P., Edgren M. R., Brunk U. T. (2010). Chelation of lysosomal iron protects against ionizing radiation. *Biochemical Journal*.

[28] Kell D. B. (2010). Towards a unifying, systems biology understanding of large-scale cellular death and destruction caused by poorly liganded iron: Parkinson's, Huntington's, Alzheimer's, prions, bactericides, chemical toxicology and others as examples. *Archives of Toxicology*.

[29] Li W., Yuan X. M., Brunk U. T. (1998). OxLDL-induced macrophage cytotoxicity is mediated by lysosomal rupture and modified by intralysosomal redox-active iron. *Free Radical Research*.

[30] Olejnicka B. T., Andersson A., Tyrberg B., Dalen H., Brunk U. T. (1999). *β*-cells, oxidative stress, lysosomal stability, and apoptotic/necrotic cell death. *Antioxidants and Redox Signaling*.

[31] Alymara V., Bourantas D., Chaidos A. (2004). Effectiveness and safety of combined iron-chelation therapy with deferoxamine and deferiprone. *Hematology Journal*.

[32] Wong C., Richardson D. R. (2003). *β*-thalassaemia: emergence of new and improved iron chelators for treatment. *International Journal of Biochemistry & Cell Biology*.

[33] Mladěnka P., Macáková K., Filipský T. (2011). In vitro analysis of iron chelating activity of flavonoids. *Journal of Inorganic Biochemistry*.

[34] Cunningham P., Afzal-Ahmed I., Naftalin R. J. (2006). Docking studies show that D-glucose and quercetin slide through the transporter GLUT1. *The Journal of Biological Chemistry*.

[35] Baccan M. M., Chiarelli-Neto O., Pereira R. M. S., Espósito B. P. (2012). Quercetin as a shuttle for labile iron. *Journal of Inorganic Biochemistry*.

[36] Li Y., Deng Y., Tang Y. (2014). Quercetin protects rat hepatocytes from oxidative damage induced by ethanol and iron by maintaining intercellular liable iron pool. *Human & Experimental Toxicology*.

[37] Kidane T. Z., Sauble E., Linder M. C. (2006). Release of iron from ferritin requires lysosomal activity. *American Journal of Physiology—Cell Physiology*.

[38] Friedman A., Arosio P., Finazzi D., Koziorowski D., Galazka-Friedman J. (2011). Ferritin as an important player in neurodegeneration. *Parkinsonism & Related Disorders*.

[39] Schafer F. Q., Buettner G. R. (2000). Acidic pH amplifies iron-mediated lipid peroxidation in cells. *Free Radical Biology and Medicine*.

[40] Terman A., Kurz T. (2013). Lysosomal iron, iron chelation, and cell death. *Antioxidants & Redox Signaling*.

[41] Yang M., Zhang M., Tahara Y. (2014). Lysosomal membrane permeabilization: carbon nanohorn-induced reactive oxygen species generation and toxicity by this neglected mechanism. *Toxicology and Applied Pharmacology*.

[42] Nilsson E., Ghassemifar R., Brunk U. T. (1997). Lysosomal heterogeneity between and within cells with respect to resistance against oxidative stress. *Histochemical Journal*.

[43] Persson H. L., Yu Z., Tirosh O., Eaton J. W., Brunk U. T. (2003). Prevention of oxidant-induced cell death by lysosomotropic iron chelators. *Free Radical Biology and Medicine*.

[44] Basu-Modak S., Ali D., Gordon M. (2006). Suppression of UVA-mediated release of labile iron by epicatechin—a link to lysosomal protection. *Free Radical Biology and Medicine*.

[45] Pourzand C., Watkin R. D., Brown J. E., Tyrrell R. M. (1999). Ultraviolet A radiation induces immediate release of iron in human primary skin fibroblasts: the role of ferritin. *Proceedings of the National Academy of Sciences of the United States of America*.

[46] Ben-Ari Z., Mor E., Azarov D. (2005). Cathepsin B inactivation attenuates the apoptotic injury induced by ischemia/reperfusion of mouse liver. *Apoptosis*.

[47] Puissant A., Colosetti P., Robert G., Cassuto J.-P., Raynaud S., Auberger P. (2010). Cathepsin B release after imatinib-mediated lysosomal membrane permeabilization triggers BCR-ABL cleavage and elimination of chronic myelogenous leukemia cells. *Leukemia*.

[48] Zhang H., Zhong C., Shi L., Guo Y., Fan Z. (2009). Granulysin induces cathepsin B release from lysosomes of target tumor cells to attack mitochondria through processing of bid leading to necroptosis. *The Journal of Immunology*.

[49] Laforge M., Limou S., Harper F. (2013). DRAM triggers lysosomal membrane permeabilization and cell death in CD4^+^ T cells infected with HIV. *PLoS Pathogens*.

[50] Jiang X., Wang X. (2004). Cytochrome C-mediated apoptosis. *Annual Review of Biochemistry*.

